# Temperament in Infancy Predicts Internalizing and Externalizing Problem Behavior at Age 5 in Children With an Increased Likelihood of Autism Spectrum Disorder

**DOI:** 10.3389/fpsyg.2022.816041

**Published:** 2022-04-18

**Authors:** Lori-Ann R. Sacrey, Lonnie Zwaigenbaum, Susan E. Bryson, Jessica A. Brian, Isabel M. Smith, Nancy Garon, Tracy Vaillancourt, Caroline Roncadin

**Affiliations:** ^1^Department of Pediatrics, University of Alberta, Edmonton, AB, Canada; ^2^Autism Research Centre, Glenrose Rehabilitation Hospital, Edmonton, AB, Canada; ^3^Dalhousie University, IWK Health Centre, Halifax, NS, Canada; ^4^Bloorview Research Institute, Toronto, ON, Canada; ^5^Department of Psychology, University of Toronto, Toronto, ON, Canada; ^6^Department of Psychology, Mount Allison University, Sackville, NB, Canada; ^7^Department of Education, University of Ottawa, Ottawa, ON, Canada; ^8^McMaster Children’s Hospital, Hamilton Health Sciences, Hamilton, ON, Canada

**Keywords:** autism spectrum disorder, parent report, increased likelihood siblings, prospective, temperament, problem behaviors

## Abstract

Differences in temperament have been linked to later mental health. Children with autism spectrum disorder (ASD) have an increased likelihood of experiencing such problems, including anxiety, depression, attention deficit/hyperactivity disorder, and oppositional defiant disorder; yet, relations between early temperament and later mental health are not well understood. In this paper, we assess the relationship between temperament in infancy and internalizing and externalizing behavior at age 5, in 178 children at an increased likelihood of being diagnosed with ASD (i.e., younger siblings of children with ASD). Temperament was assessed using the parent-reported Infant Behavior Questionnaire (IBQ) at 6 and 12 months of age and the Toddler Behavior Assessment Questionnaire-Revised (TBAQ-R) at 24 months of age. Mental health problems were assessed using the parent-reported Child Behavior Checklist (CBCL) at age 5. The data were analyzed using hierarchical multiple regressions, with individual temperament subscale scores as single predictor variables (Subscale Score) or temperament profiles using confirmatory factor analyses (Person-Centered Profile) in the first block, Autism Diagnostic Observation Schedule total severity scores at age 3 in the second block, and expressive and receptive language scores (from Mullen Scales of Early Learning) at age 3 in the third block for each model. Three main findings were: (1) 4 of 6 IBQ subscales at both 6 and 12 months significantly predicted internalizing and externalizing problems at age 5; (2) 9 and 8 of 13 TBAQ-R subscales at 24 months significantly predicted internalizing and externalizing problems, respectively, at age 5; and (3) a “sticky attention” temperament profile significantly predicted internalizing problems, whereas a “low-focused” profile significantly predicted externalizing problems, both at age 5. The results of this study support the supposition that temperament is a trans-diagnostic risk factor for later mental health conditions. Exploring temperament profiles and trajectories may illuminate early avenues for prevention in siblings of children with ASD who are at an increased likelihood of experiencing mental health problems, regardless of ASD diagnostic status.

## Introduction

Temperament refers to constitutionally based individual differences in reactivity, affect, and self-regulation (McAdams, 1995; Revelle, 1995; Rothbart and Bates, 2006). Temperament influences how a child responds to emotionally stimulating situations (Li et al., 2020) and children who have difficulty with emotional control may experience increased episodes of negative emotionality (such as anger, fear, or sadness) and increased vulnerability to mental health problems (Cole et al., 2004; Nigg, 2006; Mazefsky et al., 2013; Sayal et al., 2014; Dougherty et al., 2015; Abulizi et al., 2017). Difficulties in emotional control have been identified as a trans-diagnostic risk factor for mental health problems (Gross and John, 2003; Aldao et al., 2016; Bos et al., 2018) and may provide a potential focus to help identify children who are an increased likelihood of such problems earlier in life.

Mental health in childhood can be characterized by two overarching categories, internalizing problems, which include anxiety and depression, and externalizing problems, which include attention deficits and hyperactivity (Vaillancourt and Boylan, 2015). Research involving typically developing children has shown longitudinal associations between early temperament and later mental health problems. For example, fear and sadness in infancy and toddlerhood have been linked to internalizing symptoms in later childhood (Stifter et al., 2008; Eisenberg et al., 2009; Morales et al., 2015, 2021), whereas activity level, positive affect, and low effortful control show longitudinal associations with externalizing symptoms (Stifter et al., 2008; Eisenberg et al., 2009; Fasche and Friedlmeier, 2015; Behrendt et al., 2020; Morales et al., 2021). Because mental health problems can persist into adolescence and adulthood (Mesman and Koot, 2001; Bosquet and Egeland, 2006; Campbell et al., 2006; Dekker et al., 2007), being able to identify children who are at an increased likelihood to develop mental health problems during early childhood is critical (Harris and Handleman, 2000; Stone and Yoder, 2001).

Detecting early precursors of mental health problems in children at an increased likelihood of autism spectrum disorder (ASD) is of particular relevance. Research is mixed, with some reporting that siblings of children with ASD experience negative impacts (adjustment difficulties, depression, anxiety; Hastings, 2003; Giallo and Gavidia-Payne, 2006; Jones et al., 2020), whereas others report no adverse impacts (Hastings, 2007; Walton and Ingersoll, 2015). The inconsistent findings increase the difficulty for identifying early precursors to mental health in siblings of children with ASD and may be a result of different methodology, age at assessment, and comparison groups used. For the subset of siblings who are also diagnosed with ASD, an exploration of mental health problems is warranted. In addition to differences in social communication, repetitive behaviors, and/or restricted interests (American Psychiatric Association [APA], 2013), up to 70% of adolescents with ASD have comorbid mental health diagnoses, including social anxiety, generalized anxiety disorder, depressive disorder, and oppositional defiant disorder (Leyfer et al., 2006; Simonoff et al., 2008; Joshi et al., 2010; Salazar et al., 2015). Leyfer et al. (2006) reported that children with ASD aged 5–17 years have an average of 3 mental health diagnoses. Furthermore, children between the ages 3 and 5 experience higher rates of internalizing and externalizing symptoms compared to children with intellectual difficulties or those with average intelligence (Gadow et al., 2004, 2005; Eisenhower et al., 2005; Weisbrot et al., 2005; Hartley et al., 2008; Vaillancourt et al., 2017; Li et al., 2020). Most studies exploring mental health in children with ASD have relied on cross-sectional data. Longitudinal studies that examine the relationship between emotion control and later mental health are mainly limited to school-aged children (e.g., ages 9–15 years; Bos et al., 2018; also see review by Matson and Goldin, 2013). One exception, Ersoy et al. (2021) explored relationships between temperament at 9, 15, and 24 months and the anxiety scale on the Child Behavior Checklist (CBCL) at 36 months and reported that behavioral inhibition and effortful control are related to expression of anxiety at age 3. Thus, little is known about how mental health problems emerge during the first 5 years of life in children with ASD.

The purpose of the current paper is to examine the relationship between temperament in infancy and toddlerhood and internalizing and externalizing behaviors at age five in a sample of children who are at an increased likelihood (IL) of being diagnosed with ASD (younger siblings of children with ASD). Specifically, we aimed to identify: (1) differences in subscales on the Infant Behavior Questionnaire (IBQ; Rothbart and Derryberry, 1981) at 6 and 12 months and the Toddler Behavior Assessment Questionnaire-Revised (TBAQ-R; Rothbart et al., 2003) at 24 months between children who are diagnosed with ASD by age 5 (IL-ASD) and children who are not diagnosed with ASD by age 5 (IL-N), (2) if individual subscales on the IBQ (Rothbart and Derryberry, 1981) at 6 and 12 months and the TBAQ-R (Rothbart et al., 2003) at 24 months could predict internalizing and externalizing total problem behaviors as measured by the Child Behavior Checklist (CBCL; Achenbach and Rescorla, 2000) at age 5; and (3) if person-centered temperament profiles, created from the temperament questionnaires at 6, 12, and 24 months using latent profile analysis, could predict internalizing and externalizing total problem behaviors at age 5. Because siblings with ASD have lower scores on developmental measures, such as the Mullen Scales of Early Learning (Mullen, 1995) compared to siblings without ASD (Longard et al., 2017; Patterson et al., 2021), and Mullen scores are related to temperament profiles (Garon et al., 2022), we controlled for developmental influences on the relationship between temperament and problematic behavior by including the Receptive and Expressive Language subscales of the Mullen in our analyses. We hypothesized that: (1) children who are diagnosed with ASD by age 5 (IL-ASD) would have higher rates of negative emotionality and lower rates of positive emotionality on the temperament measures at 6, 12, and 24 months compared to the IL-N group, (2) individual subscales on the temperament measures and temperament profiles would predict internalizing and externalizing problem behaviors at age 5, and (3) developmental ability, as measured by expressive and receptive language, will affect the relationship between temperament and problem behavior.

## Materials and Methods

### Participants

Participants included children at an increased likelihood (IL) of being diagnosed with ASD (i.e., younger siblings of children with ASD) from our prospective infant sibling study that followed IL children from 6 or 12 months to 3 years of age (blinded for review). A subset of parents who attended the 3-year assessment agreed to return for a follow-up visit at age 5 (43.5%). Children were included in these analyses if they had temperament data for at least one time point (6, 12, and/or 24 months), as well as CBCL data and an assessment for ASD at age 5. Of the 409 IL children who completed their 3-year assessment, 178 (127 IL-N and 51 IL-ASD) met criteria for these analyses.

All participants were born at 36–42 weeks gestation, with birth weights greater than 2,500 grams, and no reports of birth complications or NICU stays. Participants were recruited through three major diagnostic and treatment centers in (blinded for review). Diagnosis of the older siblings was confirmed by expert clinical judgment based on DSM-IV-TR, and in most cases included the Autism Diagnostic Observation Schedule (ADOS; Lord et al., 2000) and the Autism Diagnostic Interview-Revised (ADI-R; Lord et al., 1994). None of the older siblings had a known genetic or chromosomal (e.g., fragile-X) syndrome or neurological disorder (e.g., tuberous sclerosis) that could account for the autism/ASD. The research ethics board at each institution approved this study and all families gave written informed consent prior to enrollment Participant characteristics are shown on Table 1.

**TABLE 1:** Demographic characteristics of the increased likelihood (IL) sample.

Measure	IL-N	IL-ASD	Statistic	*p*
Sex	75 boys: 52 girls	32 boys: 19 girls	*X*^2^ = 0.21	0.65
**Child age (months)**	**Mean (±SD)**	**Mean (±SD)**	**Statistic**	***P***
Age at 6-month IBQ	6.45 (0.77)	6.60 (1.07)	*Z* = –0.14	0.89
Age at 12-month IBQ	12.46 (0.91)	12.50 (0.70)	*Z* = –1.22	0.22
Age at 24-month TBAQ-R	24.43 (0.67)	24.25 (0.60)	*Z* = –1.37	0.17
Age at 3-year Ax	38.08 (2.32)	37.98 (2.73)	*Z* = –1.21	0.23
Age at CBCL/5-year Ax	65.47 (5.06)	64.23 (4.38)	*Z* = –0.59	0.56
**Parent age (years)**	**Mean (±SD)**	**Mean (±SD)**	**Statistic**	***p***
Mother age at child birth	34.17 (4.29)	34.63 (4.48)	*Z* = –0.58	0.56
Father age at child birth	36.70 (4.73)	37.49 (4.73)	*Z* = –0.53	0.59
**Measure**	**%**	**%**	**Statistic**	***P***
**Family SES**				
<20	2	0	*X*^2^ = 0.36	0.36
21 to =35	16	14		
36 to =50	35	27		
51 to =127 IL–66	47	59		
**Mothers’ ethnicity**				
Caucasian	60.62	66.67	*X*^2^ = 5.16	0.64
African	1.57	3.92		
Aboriginal	1.57	0		
Asian	3.94	3.92		
East Indian	1.58	1.96		
Southeast Asian	3.15	0		
Mixed Heritage	3.15	0		
Unknown	24.42	23.53		
**Fathers’ ethnicity**				
Caucasian	62.99	68.63	*X*^2^ = 7.93	0.44
African	0	3.92		
Aboriginal	1.57	0		
Asian	3.94	1.96		
East Indian	3.15	1.96		
Hispanic	0.80	0		
Southeast Asian	1.57	0		
Mixed Heritage	1.57	1.96		
Unknown	24.41	21.57		

*Group (ASD or Non-ASD) for statistical comparisons used blind diagnostic assessment at age 5. Socioeconomic status (SES) is based on the Hollingshead Four-Factor Index, a composite score comprised of education, occupation, sex, and marital status (Hollingshead, 1975). Hollingshead Index raw scores range from 8 to 66, with higher scores reflecting higher SES.*

*Ax, Assessment; IBQ, Infant Behavior Questionnaire; CBCL, Child Behavior Checklist; IL-ASD, Increased Likelihood sibling with ASD; IL-N, Increased Likelihood Sibling without ASD; SD, standard deviation; TBAQ-R, Toddler Behavior Assessment Questionnaire-Revised.*

### Procedure

Caregivers of children completed the IBQ at 6 and 12 months of age, the TBAQ-R at 24 months of age, and the CBCL at age 5. At age 5, each participant underwent an independent diagnostic evaluation, conducted by an expert clinician. ASD diagnoses were assigned using DSM-IV-TR criteria, based on the best judgment of the clinician (developmental pediatrician, child psychiatrist, or clinical psychologist, all with at least 10 years of diagnostic experience). Based on this assessment, participants were classified as IL children diagnosed with ASD (IL-ASD) or IL infants not diagnosed with ASD (IL-N).

### Assessments

#### Infant Behavior Questionnaire (IBQ)

The IBQ (Rothbart, 1981) is designed to assess temperament in children aged 3–12 month and has six subscales: activity level, smiling and laughing, fear, distress to limitations, soothability and duration of orienting. Items are rated on a 7-point scale ranging from 1 (never) to 7 (always), with an 8th point for does not apply. Calculation of the mean ratings on all the items in a particular scale, minus the “does not apply items” yields scaled scores. The IBQ can be completed by parents within 15 min. The IBQ is well-validated and has excellent test-retest reliability (Goldsmith and Rothbart, 1991).

#### Toddler Behavior Assessment Questionnaire—Revised (TBAQ-R)

The TBAQ-R (Goldsmith, 1996) is a reliable and well-validated measure of temperament designed for infants 18–35 months (Rothbart et al., 2003). It is comprised of 13 scales, including the original four scales from the TBAQ (Activity level, Anger/Frustration, Positive Anticipation, and Social Fear; Goldsmith, 1996). Scores were computed for each scale of the TBAQ-R by adding the scores for each item and dividing by the number of items (with reverse scoring for some items).

#### Child Behavior Checklist (CBCL)

The CBCL (Goldsmith, 1996) is designed to assess maladaptive behaviors in children between 1.5 and 5 years. Caregivers rate the frequency of internalizing and externalizing problematic behaviors on a three-point Likert scale (0 = Not True, 1 = Somewhat or Sometimes True, 2 = Very True or Often True). The Emotionally Reactive, Anxious/Depression, Somatic Complaints, and Withdrawn scales combine to yield the Internalizing Problems composite score and the Attention Problems and Aggressive behavior scales combine to yield the Externalizing Problems composite score. The scales and composite scores are converted to T-scores (*M* = 50, *SD* = 10), with a T-score of 70 and above considered to be Clinically Significant. The CBCL has adequate reliability and validity for scale scores (Achenbach and Rescorla, 2000).

#### Autism Diagnostic Observation Schedule (ADOS)

The ADOS (Lord et al., 2000) includes standardized activities and “presses,” which are used to elicit communication, social interaction, imaginative use of play materials, and repetitive behavior (Lord et al., 1989). Inter-rater reliability for the ADOS is excellent (Lord et al., 2000). The scoring algorithm was revised to optimize discrimination of ASD from other developmental disabilities and is organized into two domains, Social Affect (including Communication and Social items), and Restricted Repetitive Behaviors (Gotham et al., 2007). The original version of the ADOS was used in this study by a clinician or research staff member who had achieved research reliability. The ADOS consists of four modules, each of which is appropriate for individuals of differing language levels. To optimize comparability across modules (and thus, across language levels), we used the ADOS severity metric (Gotham et al., 2009).

#### Mullen Scales of Early Learning

The Mullen (Mullen, 1995) assesses cognitive, language, and motor abilities in children aged 0–68 months. It assesses five domains (gross motor, fine motor, expressive language, receptive language and visual reception). The Receptive and Expressive Language subscales from 36 months were used in this study as a measure of developmental ability.

### Person-Centered Temperament Profiles

Latent profile analysis (LPA) was used to identify groups of children who differed on temperament measures of attention and positive affect using scores from two scales at 6 and 12 months (Duration of Orientation and Smiling/Laughter) and three scales at 24 months (Attentional Focusing, Attentional Shifting, Positive Anticipation). To be included in the LPA analyses, at least one temperament questionnaire had to be completed at 6, 12, and/or 24 months (Garon et al., 2022). As recommended by Masyn (2013), four different specifications of the variance-covariance structure were used to select the best fit. In the simplest, most restrictive type of model, variances are fixed across different groups and covariances are set at 0. In the second type, variances are allowed to vary across groups and the covariances are set to 0. The third type sets the variance and covariances to be equal across groups. Finally, in the least restrictive model, the variance and covariances are allowed to vary across groups.

For each variance-covariance structure, the unconditional (one-profile) model was run, with the number of profiles being increased until there was no improvement in model fit (Nylund et al., 2007). Model fit was determined using several indices, including the Bayesian Information Criterion (BIC; Schwarz, 1978) and sample-size adjusted BIC (ABIC; Sclove, 1987). The Vuong-Lo-Mendell-Rubin (VLMR; Lo et al., 2001). Likelihood Ratio Test were used to evaluate model fit (Nylund et al., 2007). Entropy ranges from 0 to 1, with higher values reflecting better classification (Nylund et al., 2007). Finally, theoretical consideration is also encouraged for interpretation of the models and a smaller number of profiles is considered preferable when all other things are equal (Masyn, 2013).

Profile 1 was labeled “low focused” due to its low scores on Duration of Orientation and Attentional Focus. Profile 2 was labeled “sticky attention” due to its combination of high Duration of Orientation and low score on Attentional Shifting. Profile 3 was labeled “well-regulated” due to its high score on both Attentional Shifting and Attentional Focus (Garon et al., 2022).

### Statistical Analysis

First, demographic characteristics were compared between IL siblings with vs. without a diagnosis of ASD using chi square analyses for categorical variables (e.g., sex, ethnicity, SES), and Mann Whitney U for continuous variables (e.g., age at assessments, age of parents) for diagnostic status at age 5.

Second, Mann Whitney *U*-tests were used to compare scores on the IBQ scales at 6 and 12 months, the TBAQ-R scales at 24 months, and Internalizing and Externalizing Total Problems scores on the CBCL at age 5. The percentage of children who fell in the normal, borderline, or clinical range of the subscales of the CBCL were compared using chi-square analyses. To control for multiple testing, analyses of the temperament scales on were corrected for number of subscales on the IBQ and TBAQ and analyses of the range of clinical concern on the CBCL were corrected for the number of scales assessed using Benjamini and Hochberg corrections (Benjamini and Hochberg, 1995; method described below).

Third, hierarchical multiple regressions were completed in SPSS v. 25. Each individual subscale of the temperament questionnaire at 6 (*n* = 6), 12 (*n* = 6), and 24 (*n* = 13) months were input as single predictor variables in block 1, total severity scores on the ADOS at 36 months was included in block 2, and expressive and receptive language t-scores from the Mullen at 36 months were included in block 3 of each model. Effect sizes were calculated using Cohen’s *f*^2^ (Cohen, 1988), an appropriate metric for multiple regression models where independent and dependent variables are both continuous. Cohen’s *f*^2^ is commonly calculated using the following formula: *f*^2^ = *R*^2^/1–*R*^2^, with *f^2^* ≥ 0.02, 0.15, and 0.35 representing small, medium, and large effect sizes, respectively.

Fourth, hierarchical multiple regressions were completed with temperament profile (sticky attention vs. well-regulated; low focused vs. well-regulated) as single predictor variables in block 1, total severity scores on the ADOS at 36 months was included in block 2, and expressive and receptive language t-scores from the Mullen at 36 months were included in block 3 of each model. The addition of Mullen scores did not significantly increase the *R*^2^ value in any of the regression analyses ran, thus only the results of Models 1 and 2 are reported.

To control for multiple testing, analyses of internalizing and externalizing problem T-scores on the CBCL were corrected for number of subscales on the IBQ and TBAQ using Benjamini and Hochberg corrections (Benjamini and Hochberg, 1995). In this method, the *p*-values are ordered smallest to largest. The alpha level for each test is then set at $\frac{k*\alpha }{m}$ with k corresponding to the *p*-value’s rank (e.g., lowest *p*-value = 1) and m corresponding to the number of comparisons, which in this case was 6 for the IBQ at 6 and 12 months, and 13 for the TBAQ at 24 months. The comparisons stop once one of the *t*-tests is rejected. Thus, this method decreases the chance of false positives. Assumptions for independence of residuals was assessed using a Durbin-Watson statistic, homoscedasticity was assessed by visual inspection of a plot of studentized residuals vs. unstandardized predicted values, evidence of multicollinearity was assessed using a tolerance value of less than 0.1, and outliers were identified for values that were ± 3 standard deviations of the mean. All models passed tests of assumptions unless noted in their relevant section.

## Results

### Participant Demographics

A total of 178 IL siblings (51 with ASD and 127 without ASD) contributed to these data. There were no differences between the two groups on any of the demographic measures (see Table 1). Briefly, there was no difference for sex [*X*^2^(1) = 0.21, *p* = 0.65], nor were there group differences in exact age at assessment of temperament at 6-month (*U* = 1,046.50, *p* = 0.89), 12-month (*U* = 2247.50, *p* = 0.22), or 24-month (*U* = 2610.00, *p* = 0.17) visits, nor in 3-year diagnostic assessment (*U* = 2,646.50, *p* = 0.22), nor 5-year CBCL administration at (*U* = 3056.50, *p* = 0.56). Additionally, there were no differences between the IL siblings with and without ASD diagnoses for family SES [*X*^2^(46) = 48.91, *p* = 0.36], fathers age at birth (*U* = 2108.50, *p* = 0.59), mothers age at birth (*U* = 2097.00, *p* = 0.56), fathers ethnicity [*X*^2^(8) = 7.93, *p* = 0.44], nor mothers ethnicity [*X*^2^(7) = 5.16, *p* = 0.64].

### Attrition Analysis

Demographic characteristics and diagnostic status at age 3 were compared between the IL participants who were included in the present analyses compared to those who were not using chi squared analyses for categorical variables and Wilcoxon Signed Rank Test for continuous variables. There were no differences between completers and non-completers for mothers age at birth (*Z* = –0.11, *p* = 0.91), fathers age at birth (*Z* = –0.67, *p* = 0.50), mothers ethnicity (*X*^2^ = 128.97, *p* = 0.84), fathers ethnicity (*X*^2^ = 136.34, *p* = 0.66), family SES (*X*^2^ = 79.90, *p* = 0.30), or diagnostic status (*X*^2^ = 0.05, *p* = 0.82).

### Group Differences on Assessment Measures

Group differences for the 6, 12, and 24-month temperament subscales, temperament profiles, as well as internalizing and externalizing total problems and clinical range on CBCL are reported in Table 2. For the temperament questionnaires, there were no group differences for at 6 months; children in the IL-ASD group had lower smiling and laughter scores at 12 months (*U* = 1561.50, *p* = 0.001); and lower scores on attention shifting (*U* = 1344.00, *p* = < 0.001), inhibitory control (*U* = 1375.00, *p* = < 0.001), and soothability (*U* = 301714.50, *p* = 0.01), with higher scores on social fear (*U* = 1724.50, *p* = 0.01) at 24 months. For the temperament profiles, the IL-N group had a higher percentage of children with a well-regulated profile compared to the sticky attention profile [*X*^2^(1) = 7.35, *p* = 0.007] and low focused profile [*X*^2^(1) = 6.99, *p* = 0.008] in the IL-N group. There was no difference between the two IL groups for percentage of children in the sticky attention vs. low focused profiles [*X*^2^(1) = 7.351, *p* = 0.007].

**TABLE 2:** Group differences on measures.

Measure	IL-N Mean (± SD)	IL-ASD Mean (± SD)	*Z*	*p*
**IBQ Age 6 months**				
Activity level	4.14 (0.89)	4.09 (0.93)	–0.28	0.78
Distress to limitations	3.75 (0.76)	3.84 (0.70)	–0.32	0.73
Distress latency to stimuli	2.77 (0.89)	2.76 (0.89)	–0.02	0.98
Duration of orienting	3.07 (1.09)	3.25 (1.23)	–0.64	0.52
Smiling and laughter	5.13 (0.78)	4.67 (0.81)	–2.34	0.02
Soothing techniques	5.35 (0.87)	4.97 (0.86)	–1.73	0.08
**IBQ Age 12 months**				
Activity level	4.31 (0.89)	4.33 (1.03)	–0.05	0.97
Distress to limitations	4.04 (0.86)	3.89 (0.91)	–1.11	0.27
Distress latency to stimuli	3.13 (0.76)	3.27 (0.72)	–0.95	0.34
Duration of orienting	2.65 (0.93)	2.72 (1.07)	–0.10	0.92
Smiling and laughter	5.04 (0.84)	4.52 (0.85)	–3.44	0.001*
Soothing techniques	5.26 (0.98)	4.97 (0.99)	–1.62	0.11
**TBAQ 24 months**				
Positive anticipation	4.20 (0.84)	5.18 (0.98)	–2.26	0.02
Attention focusing	3.98 (0.79)	3.99 (0.85)	–0.27	0.79
Attention shifting	4.97 (0.91)	4.31 (0.95)	–4.09	<0.001*
Discomfort	2.32 (0.75)	2.60 (0.89)	–1.87	0.06
High pleasure	4.74 (0.80)	4.42 (0.96)	–1.67	0.10
Inhibitory control	4.12 (0.90)	3.38 (0.97)	–3.96	<0.001*
Low pleasure	5.45 (0.86)	5.21 (0.92)	–1.90	0.06
Perceptual sensitivity	3.30 (0.91)	3.29 (0.97)	–0.19	0.85
Sadness	3.35 (0.69)	3.42 (0.86)	–0.21	0.84
Soothability	5.31 (0.75)	4.95 (0.84)	–2.57	0.01*
Activity level	4.13 (0.99)	4.56 (0.90)	–2.32	0.02
Anger	3.96 (0.89)	4.09 (0.98)	–0.88	0.38
Social fear	3.69 (0.98)	4.13 (0.89)	–2.53	0.01*
**CBCL-1½ –5 age 5**				
Internalizing t-score	46.46 (12.73)	57.84 (12.18)	–5.28	<0.001*
Externalizing t-score	45.90 (12.89)	57.90 (14.16)	–4.91	<0.001*
**CBCL-1½ -5 syndrome scale range**	**% Clinical**	**% Clinical**	***X* ^2^**	***p***
Internalizing	7.87	33.33	26.07	<0.001*
Externalizing	9.45	35.29	23.39	<0.001*
Emotionally reactive	4.10	20.83	28.75	<0.001*
Anxious/Depressed	2.44	14.29	14.06	0.001*
Somatic complaints	1.63	8.16	5.79	0.06
Withdrawn	4.88	22.45	20.70	<0.001*
Sleep problems	<1	10.87	10.50	0.005*
Attention problems	4.07	22.45	20.09	<0.001*
Aggressive behavior	6.50	20.41	11.52	0.003*
**Temperament profile**	**%**	**%**	***X* ^2^**	***p***
Well-regulated	47.50	21.74	9.17	0.01
Sticky attention	25.83	39.13		
Low focused	26.67	39.13		

Note that the CBCL-1½ -5 Syndrome Scales includes three descriptive categories (normal range, borderline range, and clinical range) in the statistical analyses but we report only the percentage of children in each group falling in the clinical range for each scale. *Benjamini and Hochberg correction for IBQ at 6 and 12 months and TBAQ-R at 24 months are all q’s < 0.013 and for CBCL Clinical Range subscales are q < 0.04.

For the problem scales, children in the IL-ASD group had higher scores on both the internalizing (*U* = 1529.00, *p* = < 0.001) and externalizing (*U* = 1640.00, *p* = < 0.001) total problems scales at age 5. With respect to the clinical range, the IL-ASD group consistently had a higher percentage of children in the “clinical range” and a lower percentage of children in the “normal range” for all scales assessed.

We assessed age-related changes in IBQ scores between 6 and 12 months separately for the IL children who were diagnosed with ASD and those that were not using Wilcoxon Signed Rank Test. For the IL-ASD group, age-related differences were found only for two of six subscales; distress to latency (*Z* = –2.54, *p* = 0.01), with mean score increasing with age, and duration of orienting (*Z* = –2.59, *p* = 0.008), with mean score decreasing with age. For the IL-N group, age-related differences were found for four of six subscales; activity level (*Z* = –2.82, *p* = 0.004), distress to limitations (*Z* = –3.92, *p* = < 0.001), distress to latency (*Z* = –3.49, *p* < 0.001), which all increased with age, and duration of orienting (*Z* = –4.36, *p* < 0.001), which decreased with age.

### Six Month Temperament Predicting Problem Behavior

The addition of Mullen scores did not significantly increase the *R*^2^ value in any of the regression analyses ran, thus only the results of Models 1 and 2 are reported.

#### Internalizing Problems

Four of six temperament scales predicted internalizing problems [*Distress to Limitations*: *R*^2^ = 0.046; *F*(1, 98) = 4.68, *p* = 0.03; *f*^2^ = 0.05; *Distress Latency*: *R*^2^ = 0.057; *F*(1, 98) = 5.87, *p* = 0.017; *f*^2^ = 0.06); *Smiling and Laughter*: *R*^2^ = 0.05 *F*(1, 98) = 5.30, *p* = 0.023; *f*^2^ = 0.05; *Soothing Techniques*: *R*^2^ = 0.05 *F*(1, 97) = 5.18, *p* = 0.025; *f*^2^ = 0.05], but with small effect sizes. The addition of ADOS total severity score to the models resulted in a medium effect size for *Distress Latency* [*R*^2^ = 0.11; *F*(2, 97) = 5.88, *p* = 0.004; *f*^2^ = 0.12]. Summary of the Models for each of the temperament subscales at 6 months are presented in Table 3 and detailed results are presented in Supplementary Material.

**TABLE 3:** Predictive relationships between 5-year internalizing and externalizing problems and 6 month temperament scales.

	Internalizing problems T-score				Externalizing problem T-score			
	Model 1		Model 2		Model 1		Model 2	
Variable	B	β	B	β	B	β	B	β
Constant	43.30***		36.73***		35.77***		28.98***	
Activity level	1.30	0.094	1.80	0.13	3.08	0.19	3.59*	0.22
ADOS total severity			1.32**	0.26			1.36*	0.23
*R* ^2^	0.009		0.074		.037		.089	
*F*	0.87		6.87**		3.74		5.55*	
Constant	35.31***		30.25***		40.41***		35.61***	
Distress to limitations	3.56*	0.21	3.76*	0.22	2.14	0.11	2.31	0.12
ADOS total severity			1.27**	.25			1.21*	.21
*R* ^2^	0.046		0.11		0.012		0.035	
*F*	4.68*		6.79**		1.21		4.29	
Constant	39.53***		33.86***		44.26***		39.06***	
Distress latency	3.32*	0.24	3.68**	0.26	1.51	.093	1.84	0.11
ADOS total severity			1.36**	0.27			1.24*	0.21
*R* ^2^	0.057		0.13		0.009		0.053	
*F*	5.87*		7.81**		.86		4.50	
Constant	48.72***		43.99***		49.42***		44.90***	
Duration of orienting	–0.024	–0.002	0.14	0.012	–0.33	–0.026	–0.18	–0.014
ADOS total severity			1.23*	0.24			1.17	0.20*
*R* ^2^	0.00		0.058		0.001		.04	
*F*	0.00		5.97*		0.064		3.98	
Constant	65.95***		59.99***		72.39***		67.03***	
Smiling and laughter	–3.46*	–0.23	–3.02*	–0.20	–4.80**	–0.27	–4.41*	–0.25
ADOS total severity			1.09*	0.21			.98	0.17
*R* ^2^	0.051		0.096		0.073		0.10	
*F*	5.30*		4.82*		7.76**		2.92	
Constant	65.70***		60.46***		72.07***		67.18***	
Soothing technique	–3.24*	–0.23	–3.02*	–0.21	–4.50**	–0.27	–4.30**	–0.26
ADOS total severity			1.20*	0.24			1.12	0.19
*R* ^2^	0.051		0.11		0.073		0.11	
*F*	5.18*		5.94*		7.61**		3.84	

*p < 0.05, **p < 0.01, ***p < 0.001 for co-efficient; corrected p-value for ANOVA is < 0.037 for internalizing problems and < 0.02 for externalizing problems.

#### Externalizing Problems

Two of six temperament scales [*Smiling and Laughter*: *R*^2^ = 0.071, *F*(1, 98) = 7.76, *p* = 0.006; *f*^2^ = 0.08; *Soothing Technique*: *R*^2^ = 0.07 *F*(1, 97) = 7.61, *p* = 0.007; *f*^2^ = 0.08] predicted externalizing problems, but with small effect sizes. The addition of ADOS total severity score to the model did not increase the effect size for either subscale. Summary of the Models for each of the temperament subscales at 6 months are presented in Table 3 and detailed results are presented in the Supplementary Material.

### Twelve Month Temperament Predicting Problem Behavior

The addition of Mullen scores did not significantly increase the *R*^2^ value in any of the regression analyses ran, thus only the results of Models 1 and 2 are reported.

#### Internalizing Problems

Four of six temperament scales predicted internalizing problems [*Activity Level*: *R*^2^ = 0.056; *F*(1, 148) = 8.85, *p* = 0.003; *f*^2^ = 0.06; *Distress to Limitations*: *R*^2^ = 0.056; *F*(1, 148) = 8.80, *p* = 0.004; *f*^2^ = 0.06]; *Distress Latency*: [*R*^2^ = 0.055; *F*(1, 148) = 8.69, *p* = 0.004; *f*^2^ = 0.06; *Smiling and Laughter*: *R*^2^ = 0.069; *F*(1, 148) = 11.01, *p* = 0.001; *f*^2^ = 0.07], but with small effect sizes. The addition of ADOS total severity score to the model increased the predictive ability of two subscales [*Activity Level*: *R*^2^ = 0.13; *F*(2, 147) = 11.11, *p* < 0.001; *f*^2^ = 0.15; *Distress to Limitations*: *R*^2^ = 0.14, *F*(2, 147) = 11.65, *p* < 0.001; *f*^2^ = 0.16], with a medium effect size. Summary of the Models for each of the temperament subscales at 12 months are presented in Table 4 and detailed results are presented in Supplementary Material.

**TABLE 4:** Predictive relationships between 5-year internalizing and externalizing problems and 12 months temperament scales.

	Internalizing problems T-score				Externalizing problem T-score			
	Model 1		Model 2		Model 1		Model 2	
Variable	*B*	β	*B*	β	*B*	β	*B*	β
Constant	35.86***		29.78***		31.92***		25.92***	
Activity level	3.08**	.24	3.40***	0.26	3.87**	0.26	4.19***	0.28
ADOS total severity			1.33**	0.28			1.32**	0.23
*R* ^2^	0.056		0.13		0.066		0.12	
*F*	8.85**		12.68**		10.46**		9.02**	
Constant	37.02***		32.17***		35.62***		28.93***	
Distress to limitations	3.82**	0.24	3.96*	0.24	3.27*	0.21	3.77**	0.24
ADOS total severity			1.26**	0.26			1.35**	0.24
*R* ^2^	0.056		0.14		0.042		0.098	
*F*	8.80**		13.75***		6.49*		9.12**	
Constant	39.53***		33.86***		39.21***		34.56***	
Distress latency	3.32*	0.24	3.68**	0.26	2.97	0.16	3.10*	0.17
ADOS total severity			1.36**	0.27			1.21**	0.22
*R* ^2^	0.055		0.12		0.025		0.070	
*F*	8.69**		11.22***		3.75		7.19**	
Constant	46.19***		42.14***		46.36***		42.44***	
Duration of orienting	1.10	0.087	1.03	0.082	0.84	0.058	0.78	0.054
ADOS total severity			1.21**	0.25			1.17	0.21*
*R* ^2^	0.008		0.070		0.003		0.046	
*F*	1.14		9.79**		0.49		6.57*	
Constant	66.81***		61.80***		72.39***		67.03***	
Smiling and laughter	–3.63***	–0.26	–3.41**	–0.25	–4.80**	–0.27	–4.41*	–0.25
ADOS total severity			1.14**	0.23			0.98	0.17
*R* ^2^	0.069		0.12		0.073		0.10	
*F*	11.01***		9.16**		7.76**		2.92	
Constant	53.96***		49.45***		64.58***		59.73***	
Soothing technique	–0.94	–0.08	–0.88	–0.07	–3.27***	–0.21	–3.07*	–0.19
ADOS total severity			1.21**	0.25			1.10*	20
*R* ^2^	0.006		0.068		0.042		0.08	
*F*	0.89		9.79**		644*		6.05*	

*p < 0.05, **p < 0.01, ***p < 0.001 for co-efficient; corrected p-value for ANOVA is < 0.041 for internalizing problems and < 0.038 for externalizing problems.

#### Externalizing Problems

Three of six temperament scales [*Activity Level*: *R*^2^ = 0.066; *F*(1, 148) = 10.46, *p* = 0.002; *f*^2^ = 0.07; *Distress to Limitations*: *R*^2^ = 0.042; *F*(1, 148) = 6.49, *p* = 0.012; *f*^2^ = 0.04; *Smiling and Laughter*: *R*^2^ = 0.042, *F*(1, 148) = 6.44, *p* = 0.012; *f*^2^ = 0.04] predicted externalizing problems, but with small effect sizes. The addition of ADOS total severity score to the model did not increase the effect size. Summary of the Models for each of the temperament subscales at 12 months are presented in Table 4 and detailed results are presented in Supplementary Material.

### Two Year Temperament Predicting Problem Behavior

The addition of Mullen scores did not significantly increase the *R*^2^ value in any of the regression analyses ran, thus only the results of Models 1 and 2 are reported.

#### Internalizing Problems

Nine of thirteen temperament scales predicted internalizing problems, six with small effect sizes [*Positive Anticipation*: *R*^2^ = 0.040; *F*(1, 141) = 5.91, *p* = 0.016; *f*^2^ = 0.04; *Discomfort*: *R*^2^ = 0.077; *F*(1, 141 = 11.70, *p* = 0.001; *f*^2^ = 0.08; *Low Pleasure*: *R*^2^ = 0.052; *F*(1, 141) = 7.77, *p* = 0.006; *f*^2^ = 0.05; *Sadness*: *R*^2^ = 0.074; *F*(1, 141) = 11.22, *p* = 0.001; *f*^2^ = 0.08; *Anger*: *R*^2^ = 0.11; *F*(1, 141) = 17.31, *p* < 0.001; *f*^2^ = 0.12; *Social Fear*: *R*^2^ = 0.06; *F*(1, 141) = 8.86, *p* = 0.003; *f*^2^ = 0.06] and three with medium effect sizes [*Attention Shifting*: *R*^2^ = 0.21; *F*(1, 141 = 37.33, *p* < 0.001; *f*^2^ = 0.27; *Inhibitory Control*: *R*^2^ = 0.20; *F*(1, 141) = 34.26, *p* < 0.001; *f*^2^ = 0.25; *Soothability*: *R*^2^ = 0.19; *F*(1, 141) = 33.55, *p* < 0.001; *f*^2^ = 0.23]. The addition of ADOS total severity score to the model increased the predictive ability of five subscales, to a medium effect size for *Discomfort* [*R*^2^ = 0.13; *F*(2, 140) = 10.19, *p* < 0.001; *f*^2^ = 0.15], *Sadness* [*R*^2^ = 0.13; *F*(2, 140) = 10.63, *p* < 0.001; *f*^2^ = 0.15], and *Anger* [*R*^2^ = 0.17; *F*(2, 140) = 14.75, *p* < 0.001; *f*^2^ = 0.20], and a large effect size for *Soothability* [*R*^2^ = 0.26; *F*(2, 140) = 24.37, *p* < 0.001; *f*^2^ = 0.35] and *Attention Shifting* [*R*^2^ = 0.24; *F*(2, 140) = 22.53, *p* < 0.001; *f*^2^ = 0.32]. Summary of the Models for each of the temperament subscales at 24 months are presented in Table 5 and detailed results are presented in Supplementary Material.

**TABLE 5:** Predictive relationships between 5-year internalizing and externalizing problems and 2-year temperament scales.

	Internalizing problems T-score				Externalizing problem T-score			
	Model 1		Model 2		Model 1		Model 2	
Variable	*B*	β	*B*	β	*B*	β	*B*	β
Constant	63.62***		57.40***		64.94***		58.82***	
Positive anticipation	–3.12*	–0.20	–2.66*	–0.17	–3.53*	–0.20	–3.08*	–0.17
ADOS total severity			1.18**	.23			1.16*	0.20
*R* ^2^	0.040		0.093		0.039		0.078	
*F*	5.90*		8.12**		5.79*		5.94*	
Constant	52.93***		48.47***		53.97***		49.51***	
Attention focusing	–0.80	–0.05	–0.83	–0.05	–1.19	–0.067	–1.22	–0.068
ADOS total severity			1.29**	0.25			1.29**	0.22
*R* ^2^	0.003		0.067		0.004		0.054	
*F*	0.37		9.65**		0.63		7.29**	
Constant	77.92***		72.77***		80.21***		75.27***	
Attention shifting	–5.88***	–0.46	–5.51***	–0.43	–6.46***	–0.44	–6.11***	–0.42
ADOS total severity			0.95*	0.19			0.91*	16
*R* ^2^	0.21		0.24		0.19		0.22	
*F*	37.33***		6.32*		33.90***		4.32*	
Constant	39.40***		36.24***		43.28***		39.95***	
Discomfort	4.30***	0.28	3.92**	0.25	2.49	0.14	2.09	0.12
ADOS total severity			1.15**	0.23			1.21*	0.21
*R* ^2^	0.077		0.13		0.020		0.063	
*F*	11.70***		8.10**		2.82		6.44*	
Constant	60.43***		53.10***		54.43***		46.58***	
High pleasure	–2.29	–0.16	–1.61	–0.11	–1.11	–0.07	–0.38	–0.023
ADOS total severity			1.18**	0.23			1.26*	0.22
*R* ^2^	0.024		0.076		0.004		0.050	
*F*	3.53		7.78**		0.62		6.66*	
Constant	71.75***		67.22***		75.13***		71.33***	
Inhibitory control	–5.62***	–0.44	–5.12***	–0.40	–6.61***	–0.46	–6.19***	–0.43
ADOS total severity			0.73	0.14			0.61	0.11
*R* ^2^	0.20		0.22		0.21		0.22	
*F*	34.26***		3.40		36.88***		1.84	
Constant	67.97***		62.79***		72.90***		67.78***	
Low pleasure	–3.39**	–0.23	–3.25**	–0.22	–4.40**	–0.26	–4.26**	–0.25
ADOS total severity			1.24**	.25			1.23**	0.21
*R* ^2^	0.052		0.11		0.067		0.11	
*F*	7.77**		9.42**		10.20**		7.05**	
Constant	49.89***		45.04***		54.43***		49.64***	
Perceptual sensitivity	–0.036	–0.003	0.049	0.004	–1.56	–0.10	–1.48	–0.10
ADOS total severity			1.29**	0.25			1.27**	0.22
*R* ^2^	0.00		0.064		0.010		0.058	
*F*	0.001		9.60**		1.44		7.14**	
Constant	33.95***		30.26***		32.42***		28.74***	
Sadness	4.74***	0.27	4.55***	0.26	5.05**	0.25	4.86**	0.24
ADOS total severity			1.23**	0.24			1.22**	0.21
*R* ^2^	0.074		0.13		0.064		0.11	
*F*	11.22***		9.37**		9.67**		6.93**	
Constant	85.66***		81.21***		92.72***		88.26***	
Soothability	–6.88***	–0.44	–6.92***	–0.44	–8.33***	–0.47	–8.37***	–0.47
ADOS total severity			1.31***	0.26			1.31**	0.23
*R* ^2^	0.19		0.26		0.22		0.27	
*F*	33.55***		12.47***		38.82***		9.71**	
Constant	41.65***		38.15***		34.50***		31.12***	
Activity level	1.91	0.15	1.70	0.13	3.48**	0.24	3.28**	0.22
ADOS total severity			1.24**	0.24			1.20*	0.21
*R* ^2^	0.022		0.082		0.056		0.10	
*F*	3.19		9.06**		8.38**		6.59*	
Constant	31.48***		26.86***		28.17***		23.55***	
Anger	4.59***	0.33	4.60***	0.33	5.30***	0.33	5.31***	0.34
ADOS total severity			1.29***	0.25			1.29***	0.22
*R* ^2^	0.11		0.17		0.11		0.16	
*F*	17.31***		10.96***		17.71***		8.27**	
Constant	38.06***		36.13***		42.27***		40.17***	
Social fear	3.08**	0.24	2.57*	0.20	1.84	0.13	1.29	0.09
ADOS total severity			1.09**	0.22			1.19*	0.21
*R* ^2^	0.059		0.10		0.016		0.057	
*F*	8.86**		6.97**		2.33		6.01*	

*p < 0.05, **p < 0.01, ***p < 0.001 for co-efficient; corrected p-value for ANOVA is < 0.042 for internalizing problems and < 0.038 for externalizing problems.

#### Externalizing Problems

Eight of thirteen temperament scales predicted externalizing problems, five with small effect sizes [*Positive Anticipation*: *R*^2^ = 0.039; *F*(1, 141) = 5.79, *p* = 0.017; *f*^2^ = 0.04; *Low Pleasure: R*^2^ = 0.067; *F*(1, 141) = 10.20, *p* = 0.002; *f*^2^ = 0.10; *Sadness*: *R*^2^ = 0.064; *F*(1, 141) = 9.67, *p* = 0.002; *f*^2^ = 0.07; *Activity Level*: *R*^2^ = 0.056; *F*(1, 141) = 8.38, *p* = 0.004; *f*^2^ = 0.06; *Anger*: *R*^2^ = 0.11; *F*(1, 141) = 17.71, *p* < 0.001; *f*^2^ = 0.12] and three with medium effect sizes [*Attention Shifting: R*^2^ = 0.19; *F*(1, 141) = 33.90, *p* < 0.001; *f*^2^ = 0.23; *Inhibitory Control*: *R*^2^ = 0.21; *F*(1, 141) = 36.88, *p* < 0.001; *f*^2^ = 0.27; *Soothability*: *R*^2^ = 0.22; *F*(1, 141) = 38.82, *p* < 0.001; *f*^2^ = 0.28]. The addition of ADOS total severity score to the model increased the predictive ability of two subscales, to a medium effect size for *Anger* [*R*^2^ = 0.16; *F*(2, 140) = 13.45, *p* < 0.001; *f*^2^ = 0.19] and a large effect size for *Soothability* [*R*^2^ = 0.27; *F*(2, 140) = 25.46, *p* < 0.001; *f*^2^ = 0.37]. Summary of the Models for each of the temperament subscales at 24 months are presented in Table 5 and detailed results are presented in Supplementary Material.

### Temperament Profiles Predicting Problem Behaviors

The addition of Mullen scores did not significantly increase the *R*^2^ value in any of the regression analyses ran, thus only the results of Models 1 and 2 are reported.

#### Internalizing Problems

When compared to the well-regulated profile, the sticky attention profile significantly predicted internalizing problems, with a medium effect size [*R*^2^ = 0.13; *F*(1, 111) = 15.88, *p* < 0.001; *f*^2^ = 0.15] that did not increase with the addition of the ADOS. The low focused profile did not predict internalizing problems without the addition of ADOS total severity scores. Summary of the Models for the temperament profiles are presented in Table 6 and detailed results are presented in Supplementary Material.

**TABLE 6:** Predictive relationships between 5-year internalizing and externalizing problems and temperament profiles.

	Internalizing problems T-score				Externalizing problem T-score			
	Model 1		Model 2		Model 1		Model 2	
Variable	*B*	β	*B*	β	*B*	β	*B*	β
Constant	45.63***		41.99***		44.63***		40.90***	
Sticky attention∧	8.76***	0.35	8.08***	0.33	9.20	0.33	8.50***	0.31
ADOS total severity			1.15**	0.23			1.18**	0.21
*R* ^2^	0.14		0.18		0.11		0.15	
*F*	0.15.88***		7.22**		13.56***		5.82*	
Constant	45.63***		42.08***		44.63***		41.61***	
Low focused∧	4.25	0.17	3.64	0.15	5.39*	0.20	4.87	0.18
ADOS total severity			1.13*	0.23			0.96	0.17
*R* ^2^	0.03		0.08		0.04		0.07	
*F*	3.44		6.12*		4.65*		3.64	

∧ Compared to the Well-Regulated Profile; *p < 0.05, **p < 0.01, ***p < 0.001 for co-efficient; corrected p-value for ANOVA is < 0.038 for internalizing problems and < 0.05 for externalizing problems.

#### Externalizing Problems

When compared to the well-regulated profile, the sticky attention profile significantly predicted externalizing problems, with a small effect size [*R*^2^ = 0.11; *F*(1, 111) = 13.56, *p* < 0.001; *f*^2^ = 0.12], and the low focused profile significantly predicted externalizing problems with a small effect size [*R*^2^ = 0.04; *F*(1, 114) = 4.65, *p* = 0.033; *f*^2^ = 0.04]. With the addition of ADOS total severity scores, the predictive ability of sticky attention increased to a medium effect size [*R*^2^ = 0.15; *F*(2, 110) = 9.98, *p* < 0.001; *f*^2^ = 0.18], whereas the strength of association between the low focused profile and externalizing problems remained unchanged. Summary of the Models for the temperament profiles are presented in Table 6 and detailed results are presented in Supplementary Material.

## Discussion

In this study, we described differences on measures of temperament in infancy and toddlerhood and their developmental relationship with later internalizing and externalizing problem behaviors in a group of children at increased likelihood (IL) for ASD. Temperament questionnaires were completed at ages 6, 12 (IBQ), and 18 (TBAQ-R) months and the CBCL was completed at age 5 by the primary caregiver. There were three main findings. First, subscales on temperament questionnaires were not able to differentiate between IL sibling with and without ASD until 12 months of age. Second, individual subscales on the temperament questionnaires at all ages assessed (6, 12, and 18 months) significantly predicted internalizing and externalizing problems at age 5. Third, temperament profiles significantly predicted internalizing and externalizing problems at age 5. The results of this study support the supposition that temperament is a trans-diagnostic risk factor for mental health (Gross and John, 2003; Aldao et al., 2016; Bos et al., 2018) and may provide an early avenue for exploration in siblings of children with ASD who are at an increased likelihood of experiencing mental health problems, regardless of ASD status.

Group differences on temperament subscales did not emerge until 12 months of age, in accordance with recent reviews of the temperament literature in children with ASD (Mallise et al., 2020; Chetcuti et al., 2021). Similar to del Rosario et al. (2014) and Paterson et al. (2019), we did not find group differences at 6 months. In contrast, Zwaigenbaum et al. (2005) reported group differences, that IL-ASD had lower levels of activity at 6 months on the IBQ. The difference between our results and those of Zwaigenbaum et al. (2005) likely stems from methodological differences; the inclusion of a low likelihood group of children (LL; no family history of ASD) in the analyses, ASD categorization at 24 months of age (which arguably captures a group of children with more severe symptoms of ASD; Zwaigenbaum et al., 2016), and lack of correction for multiple comparisons in the Zwaigenbaum paper. We did find group differences at 12 months, but only on the smiling and laugher scale, with IL-ASD showing less smiling and laughing compared to IL-N siblings. Other studies exploring temperament differences at 12 or 14 months in prospective cohort of IL siblings reported differences in several different domains, including higher scores on distress to limitations, longer duration of visual orientation toward objects (Zwaigenbaum et al., 2005), less positive affect (Clifford et al., 2013; Garon et al., 2016), and were more adaptive to changes in routine (del Rosario et al., 2014) compared to IL siblings without ASD and/or LL controls. The findings across the different studies are studies were not consistent and are again likely due to methodological differences in age at ASD assessment, questionnaires used, and statistical analyses.

At 24 months, children in the IL-ASD group in this study had lower scores on attention shifting inhibitory control, and soothability, with higher scores on social fear. These results align with previous research, which consistently identified lower scores on soothability (Clifford et al., 2013; del Rosario et al., 2014; Macari et al., 2017) in children with ASD, as well as lower scores on attention shifting and inhibitory control in a cohort study that used the TBAQ to assess temperament at 24 months (Macari et al., 2017).

Subscale scores on the temperament questionnaires predicted parent-reported rates of 5-year internalizing and externalizing behavior as early as 6 months of age. For the IBQ, soothing technique at 6 months, distress to limitations and activity level at 12 months, and smiling and laughter at 6 and 12 months predicted both internalizing and externalizing behavior. For the TBAQ-R, sadness, anger, positive anticipation, low pleasure, inhibitory control, attention shifting, and soothability predicted both internalizing and externalizing behavior at 24 months. Overall, the general pattern of findings supports the hypothesis that temperament is a trans-diagnostic risk factor for mental health (Gross and John, 2003; Aldao et al., 2016; Bos et al., 2018) and are in accordance with previous research suggesting that emotional reactivity and/or expressivity, particularly negative emotions, are linked to problem behaviors (e.g., Clark et al., 1994; Eisenberg et al., 1994, 1996; Rothbart et al., 1994; Shaw et al., 1997; Lengua et al., 1998; Rothbart and Bates, 1998; Diener and Kim, 2004; Stifter et al., 2008; Burrows et al., 2016; Behrendt et al., 2020). Some authors have suggested that different negative emotions may be differentially associated with internalizing vs. externalizing behaviors (Keltner et al., 1995; Rothbart and Bates, 1998; Eisenberg et al., 2000). The current study supported such differential associations, with IBQ distress latency at 6 and 12 months and TBAQ discomfort and social fear at 24 months predicting only internalizing behavior, and TBAQ activity level at 24 months predicting only externalizing behavior. Yet, specific scales themselves were not consistently linked with either internalizing or externalizing behaviors. The majority of previous studies exploring the longitudinal relationships between temperament and problem behaviors have focused on temperament composites or author constructed profiles (Morales et al., 2021), therefore further exploration of subscales and problem behavior is warranted.

Our profiles, constructed using latent profile analysis of duration of orientation and smiling/laughter from the IBQ and attentional focusing, attentional shifting, and positive anticipation from the TBAQ-R resulted in a higher percentage of children from the IL-ASD group in the sticky attention and low focused profiles compared to the well-regulated profile. The sticky attention profile predicted internalization and externalization problem scores, whereas the low focus profile predicted externalization problem scores, only. These results are similar to those reported in Chetcuti et al. (2020) who also used latent profile analyses to identify subgroups of infants, but only using the IBQ-R, at 12 months. Three distinct subgroups also emerged, a well-regulated profile, as well as an active/negative and inhibited/low positive profile. Although the subscales that comprised our and Chetcuit’s profiles differed, the results were similar, with the active/negative profile predicting externalizing and internalizing behavior and the inhibited/low positive predicting internalizing only (as measured by the Infant-Toddler Social Emotional Assessment). Together, these findings are in agreement with literature on normative child development (Thomas et al., 1968; Thomas and Chess, 1977; Robins et al., 1996), and specifically, the longitudinal relationship between temperament and mental health (Gregory et al., 2019; Giesbrecht et al., 2020; Tang et al., 2020; Morales et al., 2021).

Differences in temperament and social-emotional behaviors are apparent in children with ASD at a very early age (see reviews by Mallise et al., 2020; Chetcuti et al., 2021) and can contribute to impairments in adaptive/functional skills (Chiang and Gau, 2016), long-term prognosis and treatment response (Horner et al., 1992; Vivanti et al., 2014), as well as increase vulnerability to mental health problems (Gadow et al., 2004; Eisenhower et al., 2005; Kagan and Fox, 2006; Leyfer et al., 2006; Nigg, 2006; Rothbart and Bates, 2006; Hartley et al., 2008; Joshi et al., 2010). Although specific temperament profiles have been associated with specific diagnoses (e.g., ADHD, Miller et al., 2019; or social anxiety disorder, Chronis-Tuscano et al., 2009), a profile for children who are or will be diagnosed with ASD has yet to be identified. Consideration of the trans-diagnostic hypothesis, supposing that temperament is a predictor of broader internalizing and externalizing symptoms, may be the proper approach for infant siblings of children with ASD for three reasons. First, this study suggests that the primary relationship exists between temperament and internalizing and externalizing behavior (i.e., ADOS scores did not consistently modify this relationship). Thus, the relationship between ASD and temperament may be driven by the presence of internalizing/externalizing behaviors. Second, although IL siblings with ASD show lower scores on developmental scales (such as Mullen, 1995) compared to IL siblings without ASD (Longard et al., 2017), and these scores are related to temperament profiles (Garon et al., 2022), the Expressive and Receptive Language subscales did not affect any of the relationships between temperamental subscales at 6, 12, and 24 months (or temperament profile) and internalizing and externalizing problem scales. Therefore, the relationship between temperament and the presence of internalizing/externalizing behaviors does not appear to be influenced by developmental ability. Third, like their siblings with ASD, non-diagnosed siblings are also at increased likelihood of mental health problems (Hastings, 2003; Meyer et al., 2011; Shivers et al., 2013; Griffith et al., 2014; Jones et al., 2020). Thus, it is critical to identify those children who display either early signs of autism, temperamental difficulties, or both to ensure that appropriate treatments can be provided (Harris and Handleman, 2000; Stone and Yoder, 2001).

Infant siblings who are later diagnosed with ASD tend to have more difficult temperamental styles. Temperament could be viewed as a modifier of ASD risk, which can act as a protective or additive factor (Mundy et al., 2007; Clifford et al., 2013; Garon et al., 2016; Chen, 2017). Compared to children with *typical* scores on temperament subscales, children who have early difficulties with attention, behavioral regulation, or reactivity may experience difficulties with social interaction and communication skill development, contributing to their risk of ASD (Zwaigenbaum et al., 2005). Furthermore, if a child has a tendency toward more negative affect, this may translate into difficulties with social interactions and learning opportunities, thereby contributing to their risk of ASD (del Rosario et al., 2014). Understanding the role of temperament as a moderator in the risk of ASD may help elucidate early differences in behavioral phenotypes that can signal a likely increased or decreased risk of the disorder.

Our findings are supportive of the trans-diagnostic hypothesis, but are not without limitations. Both the temperament and problem behavior data included in this study came from questionnaires, which may have been impacted by the common methods bias (Choi and Pak, 2005). We had a relatively small sample size and our data were comprised of responses from caregivers of children with ASD, who may have been influenced by their experience with the older sibling, affecting how the temperament or CBCL questionnaires were rated (e.g., response bias; Wetzel et al., 2016). Additionally, parents received feedback concerning their child’s performance at each visit, which may have affected how the parent scored questionnaires at later assessments. As such, ratings reported in this research context, which provided the parents of children at an increased likelihood of ASD with ongoing feedback, might be fundamentally different from parental experiences when completing questionnaires as part of a universal screen at general wellness visits. We also acknowledge that questionnaires completed as a part of this study may perform differently in infants who are at increased likelihood of ASD for reasons other than family history or idiopathic ASD. Nevertheless, the strengths of our study include a sample of 51 children diagnosed with ASD who had data collected across several timepoints in early childhood, from 6 months to 5 years.

The findings from the current study suggest that temperament is an early indicator of later internalizing and externalizing behaviors. Importantly, our results are in agreement with the trans-diagnostic hypothesis; that temperament predicts later psychopathology. Understanding the role early temperament plays in later mental health outcomes is vital for siblings of children with ASD, regardless of their own ASD status.

## Data Availability Statement

The raw data supporting the conclusions of this article will be made available by the authors, without undue reservation.

## Ethics Statement

The studies involving human participants were reviewed and approved by University of Alberta, University of Toronto, and Dalhousie University Research and Ethics Board for Human Subjects. Written informed consent to participate in this study was provided by the participants’ legal guardian/next of kin.

## Author Contributions

LZ, SB, JB, IS, NG, TV, and CR developed the research protocol, objective hypothesis, and study design. L-AS completed the analyses and wrote the first draft of the manuscript. All authors provided constructive feedback on first draft and approved final draft.

## Conflict of Interest

The authors declare that the research was conducted in the absence of any commercial or financial relationships that could be construed as a potential conflict of interest.

## Publisher’s Note

All claims expressed in this article are solely those of the authors and do not necessarily represent those of their affiliated organizations, or those of the publisher, the editors and the reviewers. Any product that may be evaluated in this article, or claim that may be made by its manufacturer, is not guaranteed or endorsed by the publisher.
