# Supplementary material for: Temperament in Infancy Predicts Internalizing and Externalizing Problem Behavior at Age 5 in Children With an Increased Likelihood of Autism Spectrum Disorder
Source: Front Psychol. 2022 Apr 18;13:816041. doi: 10.3389/fpsyg.2022.816041 (PMC9062223; doi:10.3389/fpsyg.2022.816041)
Supplement: Supplementary file 1 [file Data_Sheet_1.docx]

**Hierarchical Linear Regression – Complete Results**

**Six-Month Temperament Predicting Internalizing and Externalizing Problems**

***Internalizing Problems***

Four of six temperament scales predicted internalizing problems, but with small effect sizes. The addition of ADOS total severity score to the model resulted in a medium effect size for Distress Latency. Summary of the Models for each of the temperament subscales at 6 months are presented in Table 3. The corrected p-value for significant regressions is < .037.

***Activity Level*** ***(AL)***. Model 2 with AL and ADOS total severity score was significant (*R^2^* = .074; *F*(2, 97) = 3.90, *p* = .02; *f^2^* = .08). Model 1 with AL score alone did not predict internalizing problems T-score (*R^2^* = .009; *f^2^* = .009), but the addition of ADOS total severity score resulted in a statistically significant increase in *R^2^* (*R^2^* = .066; *F*(1, 97) = 6.87, *p* = .01)

***Distress to Limitations (DLim).*** Model 2 with DLim score and ADOS total severity score was significant (*R^2^* = .11; *F*(2, 97) = 5.88, *p* = .004; *f^2^* = .12). Model 1 with DLim alone did produce a significant *R^2^* (*R^2^* = .046; *F*(1, 98) = 4.68, *p* = .03; *f^2^* = .05) and the addition of ADOS total severity score (Model 2) resulted in a statistically significant increase in *R^2^* (*R^2^* = .062; *F*(1, 97) = 6.79, *p* = .01).

***Distress Latency (DLat).*** Model 2 with DLat score and ADOS total severity score was significant (*R^2^* = .13; *F*(2, 97) = 7.04, *p* = .001; *f^2^* = .15). Model 1 with DLat score alone did produce a significant *R^2^* (*R^2^* = .057; *F*(1, 98) = 5.87 *p* = .017; *f^2^* = .06) and the addition of ADOS total severity score (Model 2) resulted in a statistically significant increase in *R^2^* (*R^2^* = .11; *F*(1, 97) = 7.81, *p* = .006).

***Duration of Orienting (DO).*** Model 2 with DO score and ADOS total severity score was not significant (*R^2^* = .058; *F*(2, 97) = 2.99, *p* = .055; *f^2^* = .06).

***Smiling and Laughter (SL).*** Model 2 with SL score and ADOS total severity score was significant (*R^2^* = .10; *F*(2, 97) = 5.16, *p* = .007; *f^2^* = .11). Model 1 with SL score alone did produce a significant *R^2^* (*R^2^* = .05 *F*(1, 98) = 5.30 *p* = .023; *f^2^* = .05) and the addition of ADOS total severity score (Model 2) resulted in a statistically significant increase in *R^2^* (*R^2^* = .096; *F*(1, 97) = 4.82, *p* = .03).

***Soothability(ST).*** Model 2 with ST score and ADOS total severity score was significant (*R^2^* = .11; *F*(2, 96) = 5.69, *p* = .005; *f^2^* = .12). Model 1 with ST score alone did produce a significant *R^2^* (*R^2^* = .05 *F*(1, 97) = 5.18 *p* = .025;  *f^2^* = .05) and the addition of ADOS total severity score (Model 2) resulted in a statistically significant increase in *R^2^* (*R^2^* = .11; *F*(1, 96) = 5.94, *p* = .017).

***Externalizing Problems***

Two of six temperament scales (Smiling and Laughter and Soothing Technique) predicted externalizing problems, but with small effect sizes. The addition of ADOS total severity score to the model did increase the effect size. Summary of the Models for each of the temperament subscales at 6 months are presented in Table 3. The corrected p-value for significant regressions is < .02.

***Activity Level*** ***(AL)***. Model 2 with AL and ADOS total severity score was significant (*R^2^* = .089; *F*(2, 97) = 4.73, *p* = .01; *f^2^* = .10). Model 1 with AL score alone did not predict externalizing problems T-score (*R^2^* = .037; *f^2^* = .04), but the addition of ADOS total severity score resulted in a statistically significant increase in *R^2^* (*R^2^* = .089; *F*(1, 97) = 5.55, *p* = .02)

***Distress to Limitations (DLim).*** Model 2 with DLim score and ADOS total severity score was not significant (*R^2^* = .05; *F*(2, 97) = 2.77, *p* = .06; *f^2^* = .05).

***Distress Latency (DLat).*** Model 2 with DLat score and ADOS total severity score was not significant (*R^2^* = .05; *F*(2, 97) = 2.70, *p* = .07; *f^2^* = .05).

***Duration of Orienting (DO).*** Model 2 with DO score and ADOS total severity score was not significant (*R^2^* = .040; *F*(2, 97) = 2.02, *p* = .14; *f^2^* = .04).

***Smiling and Laughter (SL).*** Model 2 with SL score and ADOS total severity score was significant (*R^2^* = .10; *F*(2, 97) = 5.42, *p* = .006; *f^2^* = .11). Model 1 with SL score alone did produce a significant *R^2^* (*R^2^* = .07l, *F*(1, 98) = 7.76 *p* = .006; *f^2^* = .08) but the addition of ADOS total severity score (Model 2) did not significantly increase *R^2^* (*R^2^* = .010; *F*(1, 97) = 2.92, *p* = .09).

***Soothability (ST).*** Model 2 with ST score and ADOS total severity score was significant (*R^2^* = .11; *F*(2, 96) = 5.84, *p* = .004; *f^2^* = .12). Model 1 with ST score alone did produce a significant *R^2^* (*R^2^* = .07 *F*(1, 97) = 7.61 *p* = .007; *f^2^* = .08) but the addition of ADOS total severity score (Model 2) did not significantly increase *R^2^* (*R^2^* = .11; *F*(1, 96) = 3.84, *p* = .053).

**Twelve-Month Temperament Predicting Internalizing and Externalizing Problems**

***Internalizing Problems***

Four of six temperament scales predicted internalizing problems, but with small effect sizes. The addition of ADOS total severity score to the model increased the predictive ability of three subscales, Activity Level, Distress to Limitations, and Distress Latency, with a medium effect size. Summary of the Models for each of the temperament subscales at 12 months are presented in Table 4. The corrected p-value for significant regressions is < .041.

***Activity Level*** ***(AL)***. Model 2 with AL and ADOS total severity score was significant (*R^2^* = .13; *F*(2, 147) = 11.11, *p* < .001; *f^2^* = .15). Model 1 with AL score alone did predict internalizing problems T-score (*R^2^* = .056; *F*(1, 148) = 8.85, *p* = .003; *f^2^* = .06) and the addition of ADOS total severity score (Model 2) resulted in a statistically significant increase in *R^2^* (*R^2^* = .13; *F*(1, 147) = 12.67, *p* < .001)

***Distress to Limitations (DLim).*** Model 2 with DLim score and ADOS total severity score was significant (*R^2^* = .14, *F*(2, 147) = 11.65, *p* < .001; *f^2^* = .16). Model 1 with DLim alone did produce a significant *R^2^* (*R^2^* = .056; *F*(1, 148) = 8.80, *p* = .004; *f^2^* = .06) and the addition of ADOS total severity score (Model 2) resulted in a statistically significant increase in *R^2^* (*R^2^* = .14; *F*(1, 147) = 13.75, *p* < .001).

***Distress Latency (DLat).*** Model 2 with DLat score and ADOS total severity score was significant (*R^2^* = .12; *F*(2, 147) = 10.26, *p* < .001; *f^2^* = .14). Model 1 with DLat score alone did produce a significant *R^2^* (*R^2^* = .055; *F*(1, 148) = 8.69 *p* = .004; *f^2^* = .06) and the addition of ADOS total severity score (Model 2) resulted in a statistically significant increase in *R^2^* (*R^2^* = .12; *F*(1, 147) = 11.22, *p* = .001).

***Duration of Orienting (DO).*** Model 2 with DO score and ADOS total severity score was significant (*R^2^* = .070; *F*(2, 147) = 5.50, *p* = .005; *f^2^* = .07). Model 1 with DO score alone did not produce a significant *R^2^* (*R^2^* = .008; *f^2^* = .008), but the addition of ADOS total severity score (Model 2) resulted in a statistically significant increase in *R^2^* (*R^2^* = .070; *F*(1, 147) = 9.79, *p* = .002).

***Smiling and Laughter (SL).*** Model 2 with SL score and ADOS total severity score was significant (*R^2^* = .11; *F*(2, 147) = 10.39, *p* < .001; *f^2^* = .12). Model 1 with SL score alone did produce a significant *R^2^* (*R^2^* = .069; *F*(1, 148) = 11.01 *p* = .001; *f^2^* = .07) and the addition of ADOS total severity score (Model 2) resulted in a statistically significant increase in *R^2^* (*R^2^* = .011; *F*(1, 147) = 9.16, *p* = .003).

***Soothing Technique (ST).*** Model 2 with ST score and ADOS total severity score was significant (*R^2^* = .07; *F*(2, 147) = 5.37, *p* = .006; *f^2^* = .08). Model 1 with ST score alone did not produce a significant *R^2^* (*R^2^* = .0066; *f^2^* = .007), but the addition of ADOS total severity score (Model 2) resulted in a statistically significant increase in *R^2^* (*R^2^* = .068; *F*(1, 147) = 9.79, *p* = .002).

***Externalizing Problems***

Three of six temperament scales (Activity Level, Distress to Limitations, and Smiling and Laughter) predicted externalizing problems, but with small effect sizes. The addition of ADOS total severity score to the model did not increase the effect size. Summary of the Models for each of the temperament subscales at 12 months are presented in Table 4. The corrected p-value for significant regressions is < .038.

***Activity Level*** ***(AL)***. Model 2 with AL and ADOS total severity score was significant (*R^2^* = .12; *F*(2, 147) = 10.02, *p* < .001; *f^2^* = .14). Model 1 with AL score alone did predict externalizing problems T-score (*R^2^* = .066; *F*(1, 148) = 10.46, *p* = .002; *f^2^* = .07) and the addition of ADOS total severity score resulted in a statistically significant increase in *R^2^* (*R^2^* = .12; *F*(1, 147) = 9.02, *p* = .003).

***Distress to Limitations (DLim).*** Model 2 with DLim score and ADOS total severity score was significant (*R^2^* = .10; *F*(2, 147) = 7.98, *p* = .001; *f^2^* = .11). Model 1 with DLim score alone did produce a significant *R^2^* (*R^2^* = .042; *F*(1, 148) = 6.49 *p* = .012; *f^2^* = .04) and the addition of ADOS total severity score (Model 2) did significantly increase *R^2^* (*R^2^* = .10; *F*(1, 147) = 9.12, *p* = .003).

***Distress Latency (DLat).*** Model 2 with DLat score and ADOS total severity score was significant (*R^2^* = .07; *F*(2, 147) = 5.55, *p* = .005; *f^2^* = .08). Model 1 with DLat score alone did not produce a significant *R^2^* (*R^2^* = .025; *f^2^* = .03), but the addition of ADOS total severity score (Model 2) did significantly increase *R^2^* (*R^2^* = .07; *F*(1, 147) = 7.19, *p* = .008).

***Duration of Orienting (DO).*** Model 2 with DO score and ADOS total severity score was significant (*R^2^* = .046; *F*(2, 147) = 3.54, *p* = .031; *f^2^* = .05). Model 1 with DO score alone did not predict externalizing problems T-score (*R^2^* = .003; *f^2^* = .003), but the addition of ADOS total severity score resulted in a statistically significant increase in *R^2^* (*R^2^* = .046; *F*(1, 147) = 6.57, *p* = .011).

***Smiling and Laughter (SL).*** Model 2 with SL score and ADOS total severity score was significant (*R^2^* = .08; *F*(2, 147) = 6.35, *p* = .002; *f^2^* = .09). Model 1 with SL score alone did produce a significant *R^2^* (*R^2^* = .042, *F*(1, 148) = 6.44 *p* = .012; *f^2^* = .04) and the addition of ADOS total severity score (Model 2) did significantly increase *R^2^* (*R^2^* = .08; *F*(1, 147) = 6.05, *p* = .015).

***Soothing Technique (ST).*** Model 2 with ST score and ADOS total severity score was significant (*R^2^* = .05; *F*(2, 147) = 3.87, *p* = .023; *f^2^* = .05). Model 1 with ST score alone did not produce a significant *R^2^* (*R^2^* = .008; *f^2^* = .008), but the addition of ADOS total severity score (Model 2) did significantly increase *R^2^* (*R^2^* = .05; *F*(1, 147) = 6.57, *p* = .011).

**Two-Year Temperament Predicting Internalizing and Externalizing Problems**

***Internalizing Problems***

Nine of thirteen temperament scales predicted internalizing problems, six with small effect sizes and three with medium effect sizes (Attention Shifting, Inhibitory Control, and Soothability). The addition of ADOS total severity score to the model increased the predictive ability of four subscales, increasing to a medium effect size for Discomfort, Sadness, and Anger, and a large effect size for Soothability. Summary of the Models for each of the temperament subscales at 24 months are presented in Table 5. The corrected p-value for significant regressions is < .042.

***Positive Anticipation (PA).*** Model 2 with PA score and ADOS total severity score was significant (*R^2^* = .093; *F*(2, 140) = 7.16, *p* = .001; *f^2^* = .10). Model 1 with PA score alone did produce a significant *R^2^* (*R^2^* = .040; *F*(1, 141) = 5.91, *p* = .016; *f^2^* = .04) and the addition of ADOS total severity score (Model 2) did significantly increase *R^2^* (*R^2^* = .093; *F*(1, 140) = 8.12, *p* = .005).

***Attention Focusing (AF).*** Model 2 with AF score and ADOS total severity score was significant (*R^2^* = .067; *F*(2, 140) = 5.02, *p* = .008; *f^2^* = .07). Model 1 with AF score alone did not produce a significant *R^2^* (*R^2^* = .003), but the addition of ADOS total severity score (Model 2) did significantly increase *R^2^* (*R^2^* = .067; *F*(1, 140) = 9.65, *p* = .002).

***Attention Shifting (AS).*** Model 2 with AS score and ADOS total severity score was significant (*R^2^* = .24; *F*(2, 140) = 22.53, *p* < .001; *f^2^* = .32). Model 1 with AS score alone did produce a significant *R^2^* (*R^2^* = .21; *F*(1, 141 = 37.33, *p* < .001; *f^2^* = .27) and the addition of ADOS total severity score (Model 2) did significantly increase *R^2^* (*R^2^* = .24; *F*(1, 140) = 6.32, *p* = .013).

***Discomfort (Dis).*** Model 2 with Dis score and ADOS total severity score was significant (*R^2^* = .13; *F*(2, 140) = 10.19, *p* < .001; *f^2^* = .15). Model 1 with Dis score alone did produce a significant *R^2^* (*R^2^* = .077; *F*(1, 141 = 11.70, *p* = .001; *f^2^* = .08) and the addition of ADOS total severity score (Model 2) did significantly increase *R^2^* (*R^2^* = .13; *F*(1, 140) = 8.10, *p* = .005).

***High Pleasure (HP).*** Model 2 with HP score and ADOS total severity score was significant (*R^2^* = .08; *F*(2, 140) = 5.74, *p* = .004; *f^2^* = .09). Model 1 with HP score alone did not produce a significant *R^2^* (*R^2^* = .024; *f^2^* = .02), but the addition of ADOS total severity score (Model 2) did significantly increase *R^2^* (*R^2^* = .076; *F*(1, 140) = 7.78, *p* = .006).

***Inhibitory Control (IC).*** Model 2 with IC score and ADOS total severity score was significant (*R^2^* = .22; *F*(2, 140) =19.12, *p* < .001; *f^2^* = .28). Model 1 with IC score alone did produce a significant *R^2^* (*R^2^* = .20; *F*(1, 141) = 34.26, *p* < .001; *f^2^* = .25), but the addition of ADOS total severity score (Model 2) did not significantly increase *R^2^* (*R^2^* = .22).

***Low Pleasure (LP).*** Model 2 with LP score and ADOS total severity score was significant (*R^2^* = .11; *F*(2, 140) =8.82, *p* < .001; *f^2^* = .12). Model 1 with LP score alone did produce a significant *R^2^* (*R^2^* = .052; *F*(1, 141) = 7.77, *p* = .006; *f^2^* = .05) and the addition of ADOS total severity score (Model 2) did significantly increase *R^2^* (*R^2^* = .11; *F*(1, 140) = 9.42, *p* = .003).

***Perceptual Sensitivity (PS).*** Model 2 with PS score and ADOS total severity score was significant (*R^2^* = .064; *F*(2, 140) =4.80, *p* = .01; *f^2^* = .07). Model 1 with PS score alone did not produce a significant *R^2^* (*R^2^* = .00; *f^2^* = .00), but the addition of ADOS total severity score (Model 2) significantly increased *R^2^* (*R^2^* = .064; *F*(1, 140) = 4.80, *p* = .01).

***Sadness (Sad).*** Model 2 with Sad score and ADOS total severity score was significant (*R^2^* = .13; *F*(2, 140) =10.63, *p* < .001; *f^2^* = .15). Model 1 with Sad score alone did produce a significant *R^2^* (*R^2^* = .074; *F*(1, 141) = 11.22, *p* = .001; *f^2^* = .08) and the addition of ADOS total severity score (Model 2) significantly increased *R^2^* (*R^2^* = .13; *F*(1, 140) = 9.37, *p* = .003).

***Soothability (Soo).*** Model 2 with Soo score and ADOS total severity score was significant (*R^2^* = .26; *F*(2, 140) = 24.37, *p* < .001; *f^2^* = .35). Model 1 with Soo score alone did produce a significant *R^2^* (*R^2^* = .19; *F*(1, 141) = 33.55, *p* < .001; *f^2^* = .23) and the addition of ADOS total severity score (Model 2) significantly increased *R^2^* (*R^2^* = .26; *F*(1, 140) = 12.47, *p* = .001).

***Activity Level (AL).*** Model 2 with AL score and ADOS total severity score was significant (*R^2^* = .08; *F*(2, 140) = 6.22, *p* = .003; *f^2^* = .09). Model 1 with AL score alone did not produce a significant *R^2^* (*R^2^* = .02; *f^2^* = .02), but the addition of ADOS total severity score (Model 2) significantly increased *R^2^* (*R^2^* = .082; *F*(1, 140) = 9.06, *p* = .003).

***Anger (Ang).*** Model 2 with Ang score and ADOS total severity score was significant (*R^2^* = .17; *F*(2, 140) = 14.75, *p* < .001; *f^2^* = .20). Model 1 with Ang score alone did produce a significant *R^2^* (*R^2^* = .11; *F*(1, 141) = 17.31, *p* < .001; *f^2^* = .12) and the addition of ADOS total severity score (Model 2) significantly increased *R^2^* (*R^2^* = .17; *F*(1, 140) = 11.00, *p* = .001).

***Social Fear (SF).*** Model 2 with SF score and ADOS total severity score was significant (*R^2^* = .10; *F*(2, 140) = 8.11, *p* < .001; *f^2^* = .11). Model 1 with SF score alone did produce a significant *R^2^* (*R^2^* = .06; *F*(1, 141) = 8.86, *p* = .003; *f^2^* = .06) and the addition of ADOS total severity score (Model 2) significantly increased *R^2^* (*R^2^* = .10; *F*(1, 140) = 6.97, *p* = .009).

***Externalizing Problems***

Eight of thirteen temperament scales predicted externalizing problems, five with small effect sizes and three with medium effect sizes (Attention Shifting, Inhibitory Control, and Soothability). The addition of ADOS total severity score to the model increased the predictive ability of two subscales, increasing to a medium effect size for Anger and a large effect size for Soothability. Summary of the Models for each of the temperament subscales at 24 months are presented in Table 5. The corrected p-value for significant regressions is < .038.

***Positive Anticipation (PA).*** Model 2 with PA score and ADOS total severity score was significant (*R^2^* = .08; *F*(2, 140) = 5.96 *p* = .003; *f^2^* = .09). Model 1 with PA score alone did produce a significant *R^2^* (*R^2^* = .039; *F*(1, 141) = 5.79, *p* = .017; *f^2^* = .04) and the addition of ADOS total severity score (Model 2) did significantly increase *R^2^* (*R^2^* = .078; *F*(1, 140) = 5.94, *p* = .016).

***Attention Focusing (AF).*** Model 2 with AF score and ADOS total severity score was significant (*R^2^* = .05; *F*(2, 140) = 3.97, *p* = .021; *f^2^* = .05). Model 1 with AF score alone did not produce a significant *R^2^* (*R^2^* = .004; *f^2^* = .004), but the addition of ADOS total severity score (Model 2) did significantly increase *R^2^* (*R^2^* = .054; *F*(1, 140) = 7.29, *p* = .008).

***Attention Shifting (AS).*** Model 2 with AS score and ADOS total severity score was significant (*R^2^* = .22; *F*(2, 140) = 19.51, *p* < .001; *f^2^* = .28). Model 1 with AS score alone did produce a significant *R^2^* (*R^2^* = .19; *F*(1, 141) = 33.90, *p* < .001; *f^2^* = .23) and the addition of ADOS total severity score (Model 2) significantly increased *R^2^* (*R^2^* = .22; *F*(1, 140) = 4.32, *p* = .04).

***Discomfort (Dis).*** Model 2 with Dis score and ADOS total severity score was significant (*R^2^* = .06; *F*(2, 140) = 4.68, *p* = .011; *f^2^* = .06). Model 1 with Dis score alone did not produce a significant *R^2^* (*R^2^* = .020; *f^2^* = .02), but the addition of ADOS total severity score (Model 2) did significantly increase *R^2^* (*R^2^* = .063; *F*(1, 140) = 6.44, *p* = .012).

***High Pleasure (HP).*** Model 2 with HP score and ADOS total severity score was significant (*R^2^* = .05; *F*(2, 140) = 3.61, *p* = .028; *f^2^* = .05). Model 1 with HP score alone did not produce a significant *R^2^* (*R^2^* = .004; *f^2^* = .004), but the addition of ADOS total severity score (Model 2) did significantly increase *R^2^* (*R^2^* = .050; *F*(1, 140) = 6.66, *p* = .011).

***Inhibitory Control (IC).*** Model 2 with IC score and ADOS total severity score was significant (*R^2^* = .22; *F*(2, 140) =19.47, *p* < .001; *f^2^* = .28). Model 1 with IC score alone did produce a significant *R^2^* (*R^2^* = .21; *F*(1, 141) = 36.88, *p* < .001; *f^2^* = .27), but the addition of ADOS total severity score (Model 2) did not significantly increase *R^2^* (*R^2^* = .22).

***Low Pleasure (LP).*** Model 2 with LP score and ADOS total severity score was significant (*R^2^* = .11; *F*(2, 140) = 8.84, *p* < .001; *f^2^* = .12). Model 1 with LP score alone did produce a significant *R^2^* (*R^2^* = .067; *F*(1, 141) = 10.20, *p* = .002; *f^2^* = .10) and the addition of ADOS total severity score (Model 2) did significantly increase *R^2^* (*R^2^* = .11; *F*(1, 140) = 7.05, *p* = .009).

***Perceptual Sensitivity (PS).*** Model 2 with PS score and ADOS total severity score was significant (*R^2^* = .06; *F*(2, 140) =4.32, *p* = .015; *f^2^* = .06). Model 1 with PS score alone did not produce a significant *R^2^* (*R^2^* = .01; *f^2^* = .07), but the addition of ADOS total severity score (Model 2) significantly increased *R^2^* (*R^2^* = .058; *F*(1, 140) = 7.14, *p* = .008).

***Sadness (Sad).*** Model 2 with Sad score and ADOS total severity score was significant (*R^2^* = .11; *F*(2, 140) =8.40, *p* < .001; *f^2^* = .12). Model 1 with Sad score alone did produce a significant *R^2^* (*R^2^* = .064; *F*(1, 141) = 9.67, *p* = .002; *f^2^* = .07) and the addition of ADOS total severity score (Model 2) significantly increased *R^2^* (*R^2^* = .11; *F*(1, 140) = 6.93, *p* = .009).

***Soothability (Soo).*** Model 2 with Soo score and ADOS total severity score was significant (*R^2^* = .27; *F*(2, 140) = 25.46, *p* < .001; *f^2^* = .37). Model 1 with Soo score alone did produce a significant *R^2^* (*R^2^* = .22; *F*(1, 141) = 38.82, *p* < .001; *f^2^* = .28 ) and the addition of ADOS total severity score (Model 2) significantly increased *R^2^* (*R^2^* = .27; *F*(1, 140) = 9.71, *p* = .002).

***Activity Level (AL).*** Model 2 with AL score and ADOS total severity score was significant (*R^2^* = .10; *F*(2, 140) = 7.65, *p* = .001; *f^2^* = .11). Model 1 with AL score alone did produce a significant *R^2^* (*R^2^* = .056; *F*(1, 141) = 8.38, *p* = .004; *f^2^* = .06) and the addition of ADOS total severity score (Model 2) significantly increased *R^2^* (*R^2^* = .099; *F*(1, 140) = 6.59, *p* = .011).

***Anger (Ang).*** Model 2 with Ang score and ADOS total severity score was significant (*R^2^* = .16; *F*(2, 140) = 13.45, *p* < .001; *f^2^* = .19). Model 1 with Ang score alone did produce a significant *R^2^* (*R^2^* = .11; *F*(1, 141) = 17.71, *p* < .001; *f^2^* = .12) and the addition of ADOS total severity score (Model 2) significantly increased *R^2^* (*R^2^* = .16; *F*(1, 140) = 8.27, *p* = .005).

***Social Fear (SF).*** Model 2 with SF score and ADOS total severity score was significant (*R^2^* = .06; *F*(2, 140) = 4.21, *p* = .017; *f^2^* = .06). Model 1 with SF score alone did not produce a significant *R^2^* (*R^2^* = .016; *f^2^* = .02), but the addition of ADOS total severity score (Model 2) significantly increased *R^2^* (*R^2^* = .057; *F*(1, 140) = 6.01, *p* = .016).

**Temperament Profiles** **Predicting Internalizing and Externalizing Problems**

***Internalizing Problems***

When compared to the well-regulated profile, the sticky attention profile significantly predicted internalizing problems, with a medium effect size, and the low focused profile significantly predicted internalizing problems with a small effect size, with or without the addition of ADOS total severity scores. Summary of the Models for the temperament profiles are presented in Table 6. The corrected p-value for significant regressions is < .038.

***Sticky Attention vs. Well-Regulated Profiles.*** Model 2 with temperament profile and ADOS total severity score was significant (*R^2^* = .18; *F*(2, 110) = 12.00, *p* < .001; *f^2^* = .22). Model 1 with temperament profile alone did produce a significant *R^2^* (*R^2^* = .13; *F*(1, 111) = 15.88, *p* < .001; *f^2^* = .15) and the addition of ADOS total severity score (Model 2) did significantly increase *R^2^* (*R^2^* = .18; *F*(1, 110) = 7.22, *p* = .008).

***Low Focused vs. Well-Regulated Profiles.*** Model 2 with temperament profile and ADOS total severity score was significant (*R^2^* = .08; *F*(2, 113) = 4.86, *p* = .009; *f^2^* = .09). Model 1 with temperament profile alone did not produce a significant *R^2^* (*R^2^* = .03; *f^2^* = .03), but the addition of ADOS total severity score (Model 2) did significantly increase *R^2^* (*R^2^* = .08; *F*(1, 113) = 6.12, *p* = .015).

***Externalizing Problems***

When compared to the well-regulated profile, the sticky attention profile significantly predicted externalizing problems, with a small effect size, and the low focused profile significantly predicted externalizing problems with a small effect size. The addition of ADOS total severity scores, the predictive ability of sticky attention increased, with a medium effect size, whereas the low focused profile remained unchanged. Summary of the Models for the temperament profiles are presented in Table 6. The corrected p-value for significant regressions is < .05.

***Sticky Attention vs. Well-Regulated Profiles.*** Model 2 with temperament profile and ADOS total severity score was significant (*R^2^* = .15; *F*(2, 110) = 9.98, *p* < .001; *f^2^* = .18). Model 1 with temperament profile alone did produce a significant *R^2^* (*R^2^* = .11; *F*(1, 111) = 13.56, *p* < .001; *f^2^* = .12) and the addition of ADOS total severity score (Model 2) did significantly increase *R^2^* (*R^2^* = .15; *F*(1, 110) = 5.82, *p* = .018).

***Low Focused vs. Well-Regulated Profiles.*** Model 2 with temperament profile and ADOS total severity score was significant (*R^2^* = .07; *F*(2, 113) = 4.20, *p* = .017; *f^2^* = .08). Model 1 with temperament profile alone did produce a significant *R^2^* (*R^2^* = .04; *F*(1, 114) = 4.65, *p* = .033; *f^2^* = .04), but the addition of ADOS total severity score (Model 2) did not significantly increase *R^2^* (*R^2^* = .07; *F*(1, 113) = 3.64, *p* = .059).
